# Risk factor analysis and nomogram development for survival prediction in obese patients with severe acute pancreatitis: a retrospective study

**DOI:** 10.1186/s12876-025-04266-3

**Published:** 2025-09-26

**Authors:** Qingcheng Zhu, Xueqin Shan, Dingyu Tan, Mingfeng Lu, Yan Xu

**Affiliations:** Department of Emergency Medicine, Northern Jiangsu Peoples Hospital Affiliated to Yangzhou University, Yangzhou, 225001 China

**Keywords:** Severe acute pancreatitis, Obesity, Mortality, Prediction model, Nomogram

## Abstract

**Background:**

Currently, there is a lack of nomograms specifically designed to predict mortality risk in obese patients with severe acute pancreatitis (SAP). The aim of our study is to develop a predictive model tailored to this population, enabling more accurate anticipation of overall survival.

**Methods:**

The study included obese patients diagnosed with SAP between January 1, 2016, and December 31, 2023. Risk factors were identified through least absolute shrinkage and selection operator regression analysis. Subsequently, a novel nomogram model was developed through multivariable logistic regression analysis. An independent cohort was used for external validation. The predictive performance of the nomogram was evaluated using metrics such as the receiver operating characteristic curve, calibration curve, and decision curve analysis (DCA).

**Results:**

A total of 394 patients were included in the study, with 341 in the survival group and 53 in the deceased group. The results of the multivariate logistic analysis revealed that age, total bilirubin, blood urea nitrogen, potassium, activated partial thromboplastin time, and malignancy were independent predictors for the survival of obese patients with SAP. The nomogram exhibited superior performance compared to the Sequential Organ Failure Assessment (SOFA) score (*P* = 0.011). In the external validation cohort, the nomogram maintained good discrimination and showed improved reclassification over SOFA. Additionally, the calibration curve demonstrated satisfactory predictive accuracy, while DCA highlighted the clinical utility of the nomogram.

**Conclusion:**

Key demographic and laboratory parameters associated with the survival of obese SAP patients have been identified. These parameters were used to develop an accurate, user-friendly nomogram, potentially serving as an effective and valuable clinical tool for clinicians.

**Supplementary Information:**

The online version contains supplementary material available at 10.1186/s12876-025-04266-3.

## Background

Acute pancreatitis (AP) is a complex gastrointestinal disease with significant morbidity and mortality, characterized by a fluctuating course that is often difficult to predict in the early stages [1]. While most AP patients experience a mild course, about 20% develop severe acute pancreatitis (SAP) [2], which involves pancreatic necrosis and organ failure, with a fatality rate ranging from 30 to 50% [3]. Therefore, early diagnosis of SAP is crucial for reducing mortality in AP.

The global prevalence of obesity is steadily rising, affecting approximately 20% of intensive care unit (ICU) patients [4]. Body mass index (BMI) is used to define obesity, calculated as weight (kg) divided by height squared (m²) [5]. Despite varying definitions of obesity, globally over 35% of adults are overweight (BMI > 25 kg/m²) and more than 10% are classified as obese (BMI > 30 kg/m²) [6]. Obesity is a well-established risk factor for worse outcomes in AP [7]. The chronic low-grade inflammation associated with obesity may increase susceptibility to both extrinsic and intrinsic factors that lead to AP [8]. Obese AP patients exhibit elevated levels of inflammatory markers [9]. Additionally, obesity and a high-fat diet promote inflammation by inhibiting autophagy [10].

Various scoring models, including the Sequential Organ Failure Assessment (SOFA), Acute Physiology and Chronic Health Evaluation II (APACHE II), and Ranson, have been employed in recent years for stratifying AP risk [11]. Despite their widespread use, these models have faced criticism due to their complexity, inconvenience, and limited accuracy [12]. Recent investigations indicate that C-reactive protein [13], red blood cell distribution width [14], and D-dimer levels [15] could potentially predict hospital mortality in SAP. However, differing threshold values for these markers across studies have resulted in inconsistencies.

A nomogram is a useful mathematical tool for predicting specific outcomes, such as disease progression or mortality, based on critical parameters [16]. Previous studies have developed nomograms to predict in-hospital mortality in SAP using critical care databases [17]. However, these studies were limited by small sample sizes and a lack of external validation. Despite this, there is a lack of systematic models specifically designed for obese patients with SAP to predict in-hospital mortality.

This study aimed to determine the risk factors associated with hospital mortality in obese patients suffering from SAP and to create a user-friendly and effective nomogram. The proposed model is designed to assist clinicians in tailoring personalized intervention strategies, ultimately improving patient outcomes.

## Methods

### Study design and eligibility

This retrospective study was carried out in a 52-bed ICU at a tertiary teaching hospital in Jiangsu Province, China. All patients were directly admitted to the ICU from the emergency department, and no patients were initially treated in a general ward prior to ICU admission. The study adhered to the ethical guidelines set forth by the revised Declaration of Helsinki. Approval was obtained from the Institutional Ethics Committee of Northern Jiangsu People’s Hospital (No. 20231204). Written informed consent was waived due to the retrospective nature of the study.

The study included obese patients diagnosed with SAP between January 1, 2016, and December 31, 2023. AP was diagnosed if at least two of the following three criteria were met: abdominal pain, elevated levels of amylase or lipase exceeding three times the upper limit of normal, or abdominal imaging findings consistent with AP, based on the 2012 revised Atlanta criteria [18]. SAP was defined as the presence of persistent organ failure lasting more than 48 h. Organ failure was defined as a Marshall score of ≥ 2, indicating that at least one organ system (respiratory, cardiovascular, renal) must be affected.

The inclusion criteria for this study were: participants needed to be obese (BMI ≥ 30 kg/m²), at least 18 years old, and have complete laboratory parameters and clinical data available within 24 h of admission. Exclusion criteria included pregnancy, a history of pancreatic surgery, and a history of abdominal trauma. All relevant data were collected within the first 24 h following ICU admission.

An independent external validation cohort was constructed using data from obese SAP patients admitted to the emergency intensive care unit (EICU) of our hospital from January 1, 2020, to December 31, 2023. The same inclusion and exclusion criteria were applied. The nomogram developed from the training cohort was applied to this external dataset. Model performance was evaluated using receiver operating characteristic (ROC) curve, calibration curve, and decision curve analysis (DCA).

### Data collection

Data were retrieved from electronic medical records, which included demographic information such as sex, age, height, weight, and medical history. Admission vital signs were also documented, specifically respiratory rate (RR), heart rate (HR), systolic blood pressure (SBP), and diastolic blood pressure (DBP). The SOFA scores were evaluated at the time of entry. Laboratory parameters analyzed included red blood cell, white blood cell, platelet, neutrophil, and lymphocyte counts. Additionally, hemoglobin, hematocrit, total bilirubin, alanine aminotransferase, aspartate aminotransferase, alkaline phosphatase, lactate dehydrogenase, serum sodium, potassium, calcium, glucose, creatinine, blood urea nitrogen (BUN), albumin, prothrombin time (PT), activated partial thromboplastin time (APTT), and the international normalized ratio (INR) were assessed. Furthermore, it was noted whether patients required invasive ventilatory support or the administration of vasopressors upon hospital admission.

### Statistical analysis

The Kolmogorov-Smirnov test was used to test the normal distribution for measurement data. Normally distributed data were expressed as means ± standard deviation, and the skewed distributed data was reported as medians (quartiles). Group comparisons were conducted using t-tests or Mann-Whitney U tests, with categorical data analyzed via chi-square or Fisher’s exact tests. The least absolute shrinkage and selection operator (LASSO) regression analysis identified key predictors of mortality, which were then incorporated into a multivariate logistic regression model to develop a predictive nomogram.

The area under the ROC curve (AUC) and the C-index were employed to evaluate the predictive accuracy of the nomogram, while a calibration curve assessed the alignment between predicted and observed outcomes. The net reclassification improvement (NRI) was utilized to compare the predictive accuracy of the nomogram with that of the SOFA scores, and the integrated discrimination improvement (IDI) measured the enhancement in model performance. DCA was conducted to determine the clinical relevance of the model. Statistical analyses were performed using R (version 3.6.1) and SPSS (version 24.0), with a significance threshold established at *P* < 0.05.

## Results

### Baseline characteristics and outcomes

**Fig. 1:**
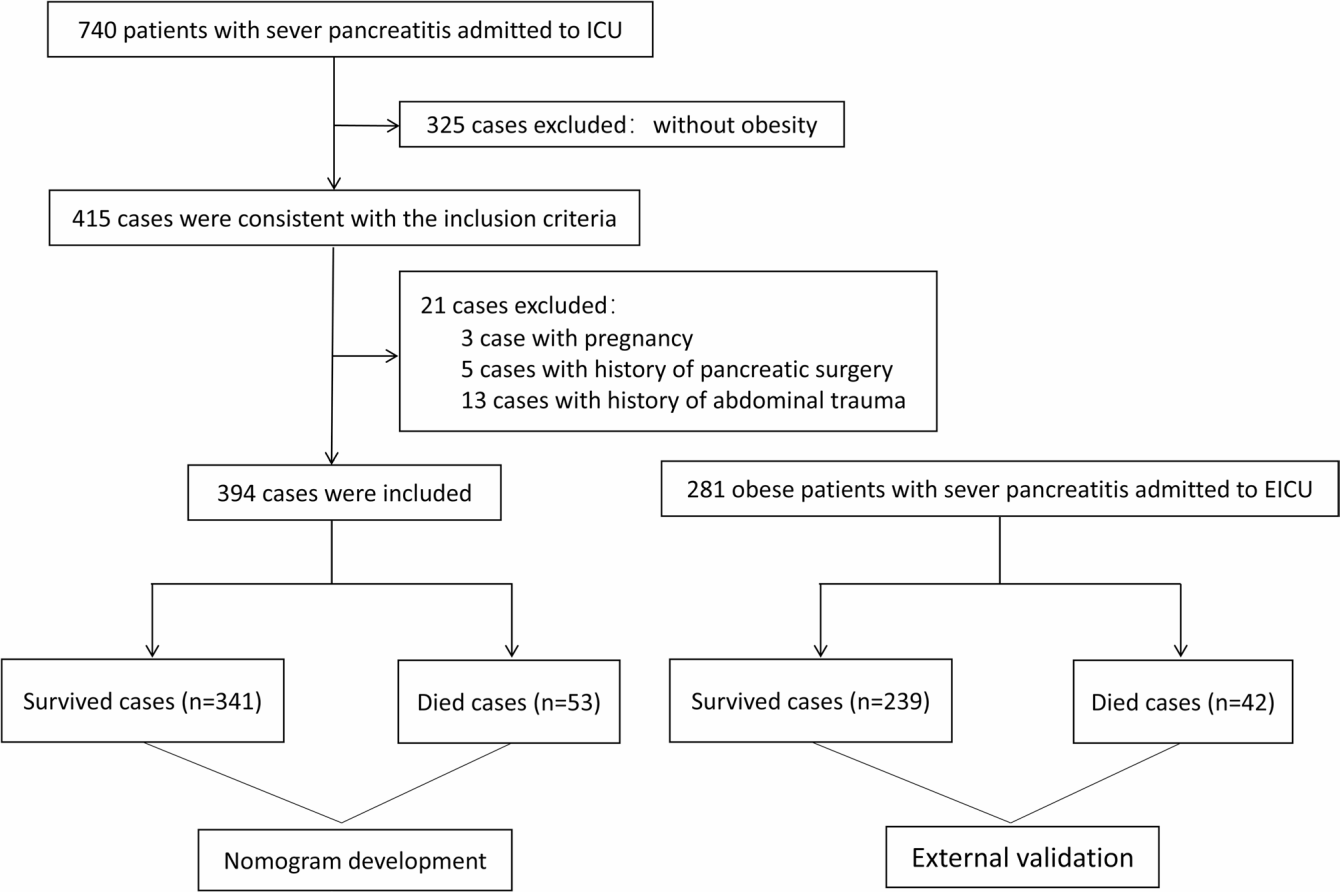
Flow chart of patient enrollment. ICU, intensive care unit; EICU, emergency ICU

Of the 415 obese patients who met the inclusion criteria during the study period, 21 were excluded for the following reasons: 3 due to pregnancy, 5 with a history of pancreatic surgery, and 13 with a history of abdominal trauma. Ultimately, 394 patients were included in the analysis, with 341 in the survival group and 53 in the deceased group (Fig. 1). The observed in-hospital mortality rate was 13.5%.

Significant differences between the two groups were observed in the following variables: age, presence of malignant tumors, SOFA score, red blood cell count, hemoglobin levels, aspartate aminotransferase, potassium, creatinine, BUN, albumin, INR, PT, APTT, and vasopressor use (*P* < 0.05). All other baseline clinicopathological characteristics were comparable, as detailed in Table 1. Among the 26 patients with malignancies, 15 were in the survival group and 11 in the deceased group. All tumors were extra-pancreatic solid malignancies, most commonly lung cancer (*n* = 8), gastric cancer (*n* = 8), colorectal cancer (*n* = 6), and hepatocellular carcinoma (*n* = 4). The detailed distribution is summarized in Supplementary Table S1.

**Table 1:** Baseline characteristics of selected patients

Characteristics	Survived(*n* = 341)	Died(*n* = 53)	*P* value
Male, n (%)	211(61.9)	28(52.8)	0.228
Age, years	65(54–76)	54(41–65)	< 0.001
Height (cm)	170(163–178)	165(160–175)	0.074
Weight (kg)	96.8(86.0-109.1)	92.9(82.6-108.8)	0.262
Comorbidities, n (%)			
COPD	80(23.5)	11(20.7)	0.664
Coronary artery disease	21(6.2)	5(9.4)	0.372
Hypertension	189(55.4)	28(52.8)	0.724
Cholelithiasis	4(1.2)	0(0)	0.428
Malignant tumor	15(4.4)	11 (20.8)	< 0.001
Diabetes mellitus	62(18.2)	14(26.4)	0.158
Vital signs			
Respiratory rate (/min)	21(16–26)	23(19–28)	0.100
Heart rate (/min)	100(84–118)	106(87–118)	0.418
Systolic blood pressure (mmHg)	123(108–143)	121(102–146)	0.315
Diastolic blood pressure (mmHg)	71(59–84)	65(52–81)	0.069
SOFA	5(3–9)	11(8–13)	< 0.001
Laboratory data			
Red blood cell (*10^12^)	3.82(3.13–4.43)	3.29(2.62–4.32)	0.047
White blood cell (*10^9^)	13.7(9.2–18.7)	14.6(10.0-21.3)	0.337
Platelet (*10^9^)	216 (159–309)	202(121–304)	0.173
Neutrophil (*10^9^)	12.7(8.5–14.9)	11.6(7.3–15.6)	0.411
Lymphocyte (*10^9^)	1.27(0.76–1.38)	1.15(0.48–1.41)	0.211
Hemoglobin (g/L)	114(95–136)	100(84–127)	0.016
Hematocrit (%)	35(29–40)	32(27–39)	0.056
Total bilirubin (mg/dL)	1.1(0.8–1.8)	2.1(1.1–3.8)	0.183
Alanine aminotransferase (U/L)	48(23–157)	46(31–118)	0.682
Aspartate aminotransferase (U/L)	68(33–188)	101(53–255)	0.041
Lactate dehydrogenase (U/L)	505(281–601)	407(319–797)	0.148
Sodium (mmol/L)	138(135–141)	137(131–142)	0.397
Potassium (mmol/L)	4.1(3.7–4.6)	4.5(4.1–5.6)	< 0.001
Chloride(mmol/L)	103(99–107)	103(96–109)	0.362
Calcium (mmol/L)	2.02(1.88–2.15)	2.03(1.83–2.13)	0.525
Glucose (mmol/L)	7.6(6.1–11.9)	8.2(5.5–10.7)	0.786
Creatinine (mg/dL)	1.2(0.8–1.8)	1.9(1.2–2.7)	< 0.001
Blood urea nitrogen (mmol/L)	19(13–30)	38(24–66)	< 0.001
Albumin (g/dL)	3.0(2.6–3.4)	2.9(2.3–3.2)	0.047
Triglyceride (mg/dL)	237 (115–255)	255(153–255)	0.475
Amylase (U/L)	160(71–176)	165(94–580)	0.180
International normalized ratio (seconds)	1.3(1.2–1.5)	1.4(1.2-2.0)	< 0.001
Prothrombin time (seconds)	14.4(13.0–17.0)	15.8(14.0-21.7)	< 0.001
Activated partial thromboplastin time (seconds)	30.2(26.9–35.0)	32.1(27.4–51.6)	0.017
Invasive ventilator	166(48.7)	31(58.5)	0.184
Vasopressor	115(33.7)	31(58.5)	0.001

*COPD* Chronic obstructive pulmonary disease, *SOFA* Sequential Organ Failure Assessment

### Construction of a predictive nomogram

**Fig. 2:**
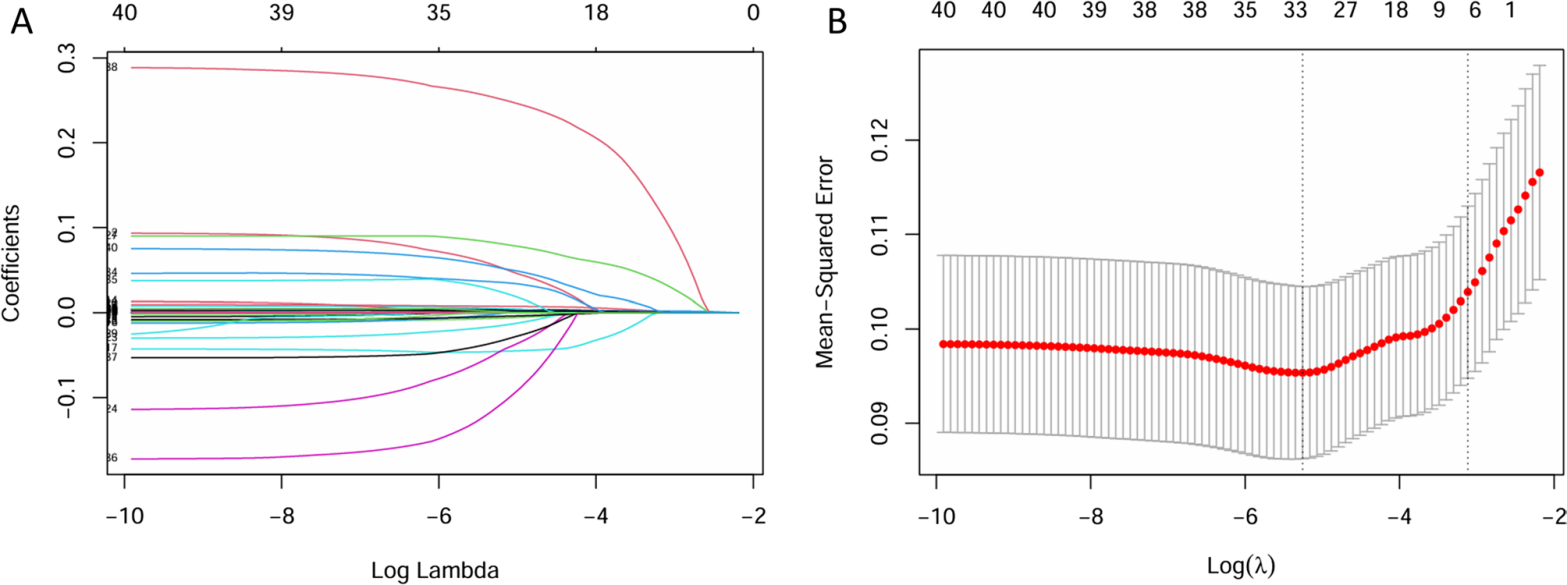
Identification of the risk factors of hospital mortality by LASSO regression. **A** Least absolute shrinkage and selection operator coefficient profiles of variables. **B** The cross-validation curves for LASSO regression are presented, with the y-axis representing binomial deviation. The dashed line on the left indicates the log(λ) at which the model deviation is minimized, while the dashed line on the right represents the log(λ) at the minimum model deviation plus one standard error. We selected the log(λ) and the corresponding variables associated with the dashed line on the right

**Fig. 3:**
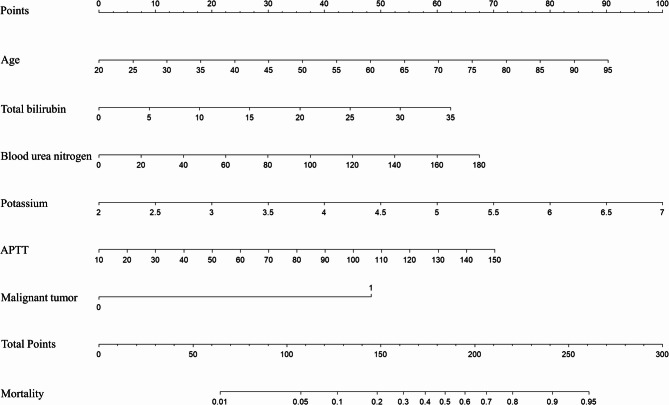
Predictive nomogram for the probability of hospital mortality in obese patients with severe acute pancreatitis

The variables identified were further examined using LASSO binary logistic regression, with lambda selected based on the 1-standard-error criterion (Figure 2A and 2B). Six independent risk factors for mortality were identified: age, total bilirubin, BUN, potassium, APTT, and malignancy, distinguishing survivors from non-survivors. A subsequent multivariate logistic regression analysis was performed on these variables, resulting in the development of a multi-factor risk model through the stepwise backward selection method (Table2). These six factors were then combined into a novel predictive nomogram (Figure 3).

**Table 2:** Multivariate logistic regression analysis of the predictors for hospital mortality

Variables	OR	95%CI	*P* value
Age	3.179	1.742–5.801	< 0.001
Total bilirubin	1.171	1.020–1.343	0.025
Blood urea nitrogen	1.353	1.067–1.715	0.013
Potassium	1.849	1.349–2.535	< 0.001
Activated partial thromboplastin time	1.189	1.016–1.390	0.031
Malignant tumor	6.396	2.258–18.112	0.001

### Evaluation and validation of the nomogram

**Fig. 4:**
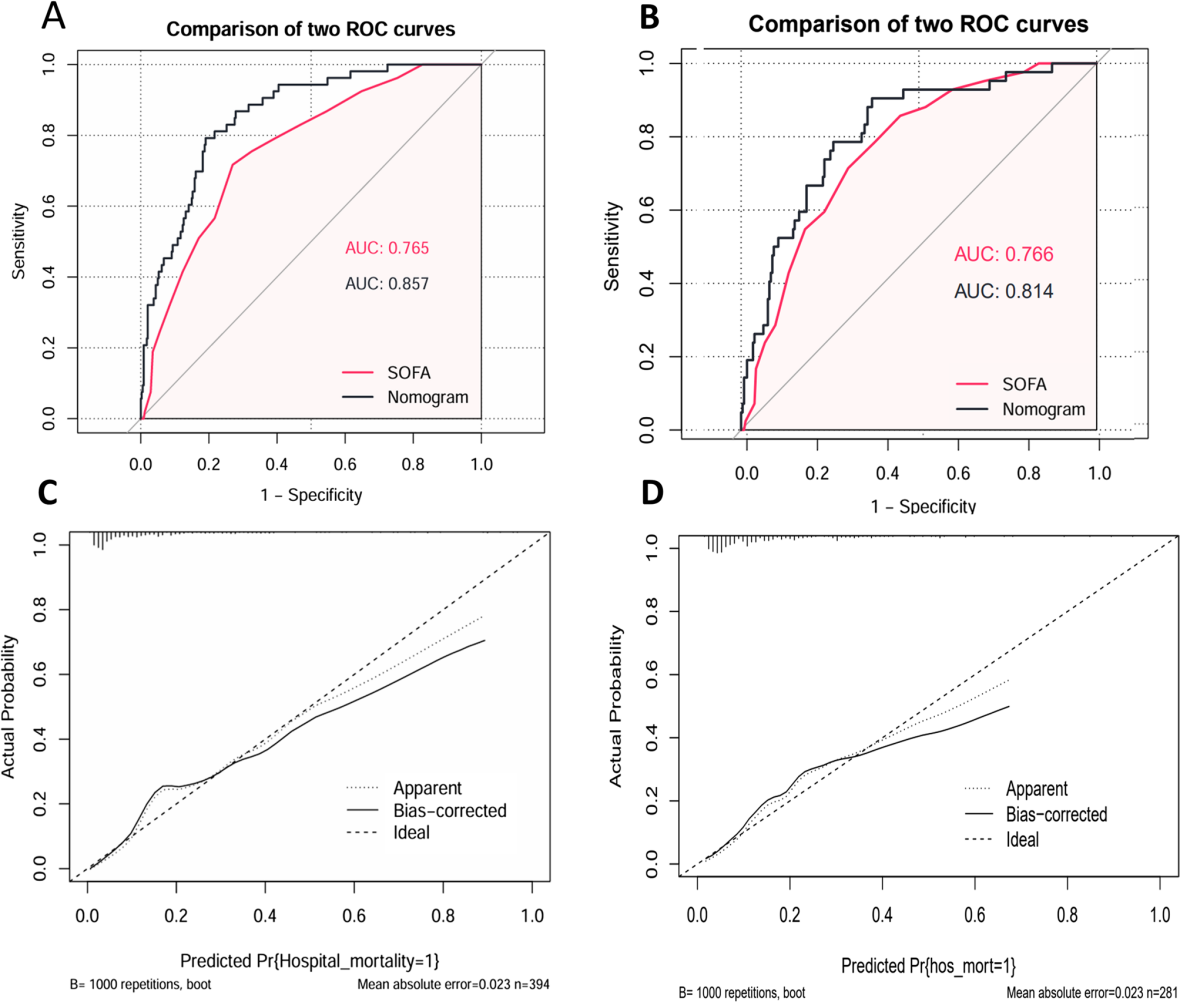
Performance of the nomogram in the training and external validation cohorts

The ROC curve analysis was utilized to assess the diagnostic accuracy of the nomogram model. Visual inspection of the AUC revealed that the nomogram outperformed the SOFA score. The AUC values for the nomogram and the SOFA score were 0.857 and 0.765, respectively, with statistically difference (*P* = 0.011, Figure 4A). The parameters corresponding to the ROC curves at the nomogram’s cut-off point are detailed in Table 3.

To internally validate the model’s performance, the bootstrapping technique was applied. The calibration plot visually illustrates a strong concordance between the predicted and observed mortality rates (Figure 4C). In this study, the IDI for the nomogram was 0.141 (95% CI: 0.068 to 0.214) compared to the SOFA score, with difference reaching statistical significance (*P* < 0.001). Furthermore, the NRI of the nomogram was significantly higher than that of the SOFA score (*P *= 0.002, Table 3).

**Fig. 5:**
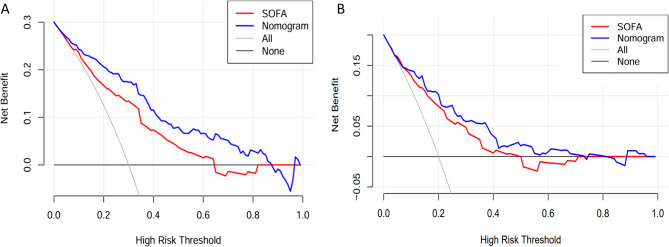
Decision curve analysis of the nomogram and SOFA score in the training (**A**) and external validation (**B**) cohorts. SOFA, sequential organ failure assessment

Figure 5A illustrates the net benefit of using the nomogram model and the SOFA score. DCA revealed that the nomogram offered superior net benefit across a broad and clinically relevant range of threshold probabilities. Moreover, clinical interventions guided by the nomogram demonstrated higher net benefit than those based on the SOFA score when the threshold probability ranged from 0.1 to 0.9.

**Table 3:** Comparison of the nomogram model and SOFA for predicting the hospital mortality

Variables	C-index (95%CI)	Sensitivity	Specificity	IDI	NRI
Development set					
Nomogram	0.857(0.809–0.906)	0.792	0.809	0.141	0.192
SOFA	0.765(0.701–0.830)	0.717	0.730	-	-
External validation set					
Nomogram	0.814(0.785–0.892)	0.905	0.632	0.082	0.385
SOFA	0.766(0.712–0.856)	0.714	0.699		

*SOFA* Sequential Organ Failure Assessment, *IDI* integrated discrimination improvement, *NRI* net reclassification improvement

### External validation performance

The baseline characteristics of the development and external validation cohorts are summarized in Supplementary Table S2. Most variables were comparable between the two cohorts, although differences were observed in comorbidities such as COPD, coronary artery disease, and diabetes mellitus.

In the external validation cohort (n=281), the nomogram showed good discrimination with an AUC of 0.814, compared to 0.766 for the SOFA score (P = 0.272) (Figure 4B). The calibration curve demonstrated satisfactory agreement between the predicted and observed mortality (Figure 4D). The DCA curve indicated that the nomogram provided greater net benefit than the SOFA score across a wide range of threshold probabilities (Figure 5B). Additionally, the IDI was 0.082 (P = 0.048), and the NRI was 0.385 (P = 0.019), confirming superior reclassification performance of the nomogram (Table 3).

## Discussion

In recent decades, the incidence of AP has steadily risen, coinciding with the increasing prevalence of obesity [19]. Gallstone disease accounts for 35-40% of AP cases globally [8]. While obesity is a well-known risk factor for cholelithiasis and biliary sludge, it also independently contributes to a more severe progression of AP [20]. To the best of our knowledge, this is the first study to develop a comprehensive nomogram specifically designed for obese patients with SAP. In this study, we identified six independent prognostic factors for in-hospital mortality in this population: age, total bilirubin, BUN, potassium, APTT, and malignancy. The nomogram demonstrated exceptional predictive accuracy for in-hospital mortality and showed superior discrimination and clinical utility compared to the conventional SOFA system. In addition, we externally validated the nomogram using an independent cohort from the EICU, confirming the model’s generalizability and robustness.

Numerous studies have reported increased mortality and morbidity in obese patients with AP, establishing obesity itself as a prognostic factor in AP [21]. In obesity, adipose tissue can exceed 30% of total body weight, with visceral fat comprising more than 3% [22]. The accumulation of visceral fat in and around the pancreas exacerbates the severity of AP, leading to poorer outcomes [19]. This can manifest as early hypocalcemia or later as organ failure. The resulting damage to visceral fat, known as fat necrosis, is a key component of radiographic criteria for assessing the severity of AP, including the revised Atlanta classification [7].

The current clinical tools used to assess the severity and prognosis of SAP include the SOFA score, APACHE II score, and Ranson score. SOFA and APACHE II are commonly employed in ICUs for predicting disease severity and mortality [23]. However, both SOFA and APACHE II have the drawback of depending on a variety of variables that are not typically available at the time of general hospital admission [24]. In contrast, the Ranson score requires 48 hours of inpatient monitoring, which can delay timely triage and treatment decisions [25]. Our study identifies a set of six easily obtainable clinical and laboratory variables to construct a simple, interpretable, and clinically practical model, which can be applied early upon ICU admission. In addition to age and malignancy, the other risk factors it incorporates can be adjusted through prompt and aggressive medical intervention, which is vital for improving patient prognosis.

Age has long been established as a key predictor of poor outcomes in AP, as reflected in both the APACHE II and Ranson scores [26]. This connection is likely due to the increased prevalence of comorbidities with advancing age [27]. Elderly patients, who often have reduced organ reserve, experience a persistent inflammatory state, contributing to a higher risk of complications [28]. Our study revealed a hospital mortality rate of 13.5% for obese patients with SAP, aligning with previously published data [29]. Additionally, age was identified as an independent risk factor, highlighting the importance of vigilant monitoring for elderly patients due to the higher risk of poor outcomes associated with advancing age.

Total bilirubin was found to be a key predictor of mortality in AP, a result supported by numerous prior studies [30]. Previous research has demonstrated that bile duct obstruction in AP can hinder bile excretion, causing bilirubin to accumulate in liver cells [31]. This accumulation disrupts normal liver cell metabolism, leading to degeneration, necrosis, and impaired liver function. Elevated bilirubin levels are strongly linked to poor patient outcomes [32]. Additionally, our study identified APTT as a critical predictor of mortality in SAP. This may be attributed to the frequent occurrence of hypocalcemia in SAP patients, which can result in coagulation disorders [33]. As such, regular monitoring of APTT in SAP patients is essential for managing their condition effectively.

BUN levels have been recognized as a reliable early predictor of SAP, with elevated BUN levels (>10.745 mmol/L) being strongly associated with an increased risk of ICU admission [34]. BUN is synthesized in the liver and excreted by the kidneys. Although traditionally studied in the context of renal diseases, recent research has demonstrated that both BUN concentrations and their fluctuations can effectively predict the severity of AP and associated mortality with high sensitivity [35]. In the study by Koutroumpakis, an increase in BUN within the first 24 hours of admission was identified as the most accurate predictor of persistent organ failure and pancreatic necrosis [32], surpassing the predictive value of other laboratory parameters.

Like BUN, potassium is also an indicator of SAP severity. Potassium is a highly abundant in intracellular fluids and plays a crucial role in intracellular metabolic activities [36]. Studies show that both low and high potassium levels are associated with increased all-cause mortality in critical ill patients [37]. In line with Pan's research findings, it has been observed that high serum potassium levels independently increase the risk of mortality in obese patients with pancreatitis [38]. Hence, it is crucial to ensure that potassium levels remain within normal range.

To enhance the generalizability of the model, we performed external validation using a separate cohort from the EICU. Despite differences in baseline characteristics, the nomogram maintained good discriminative ability and net clinical benefit, and outperformed the SOFA score in reclassification metrics. These findings support the robustness and potential applicability of our model in broader clinical settings.

However, our study has several limitations. Firstly, it is retrospective in nature, which raises the possibility of selection and detection bias. To enhance the level of evidence, prospective studies are warranted. Secondly, although we performed external validation using an independent cohort from the EICU within the same institution, the model has not yet been tested across multiple centers or diverse geographic populations. Thirdly, although we made efforts to adjust for confounding factors using multivariate logistic regression analysis, residual confounding from unknown or unmeasured covariates may still be present.

## Conclusion

A nomogram model was developed for obese patients with SAP, incorporating clinical and laboratory parameters measured upon admission to predict hospital mortality accurately. This tool shows promise in aiding clinicians to tailor individualized treatment plans for these patients, ultimately enhancing patient outcomes, conserving medical resources and costs, and facilitating early recovery.

## Supplementary Information

Supplementary Material 1.

## Data Availability

The analyzed datasets used in this study and all analysis output reports are available upon reasonable request from the corresponding author.

## Abbreviations

AP: Acute pancreatitis
SAP: Severe acute pancreatitis
ICU: Intensive care unit
BMI: Body mass index
SOFA: Sequential Organ Failure Assessment
APACHE II: Acute Physiology and Chronic Health Evaluation II
EICU: Emergency intensive care unit
RR: Respiratory rate
HR: Heart rate
SBP: Systolic blood pressure
DBP: Diastolic blood pressure
BUN: Blood urea nitrogen
PT: Prothrombin time
APTT: Activated partial thromboplastin time
INR: International normalized ratio
LASSO: Least absolute shrinkage and selection operator
AUC: Area under the receiver operating characteristic curve
NRI: Net reclassification improvement
IDI: Integrated discrimination improvement
DCA: Decision-curve analysis
ROC: Receiver operating characteristic
