# Supplementary material for: Risk factor analysis and nomogram development for survival prediction in obese patients with severe acute pancreatitis: a retrospective study
Source: BMC Gastroenterol. 2025 Sep 26;25:651. doi: 10.1186/s12876-025-04266-3 (PMC12465976; doi:10.1186/s12876-025-04266-3)
Supplement: Supplementary file 1 — Supplementary Material 1. [file 12876_2025_4266_MOESM1_ESM.docx]

**Table S1** Distribution of malignant tumor types in the survival and deceased groups.

| **Tumor Type** | **Survival Group (n = 15)** | **Deceased Group (n = 11)** |
| --- | --- | --- |
| **Lung cancer** | 4 | 4 |
| **Gastric cancer** | 5 | 3 |
| **Colorectal cancer** | 3 | 3 |
| **Hepatocellular carcinoma** | 3 | 1 |

**Table S2** Baseline characteristics of patients in the development and external validation cohorts.

| Characteristics | Development set（n=394） | External validation set（n=281） | *P* value |
| --- | --- | --- | --- |
| Male, n (%) | 239(60.7) | 174(61.9) | 0.801 |
| Age, years | 55 (42-67) | 56 (44-71) | 0.162 |
| Height (cm) | 169 (163-178) | 170 (163-181) | 0.686 |
| Weight (kg) | 96.4(85.0-109.0) | 94.8(83.2-109.3) | 0.535 |
| Comorbidities, n (%) |  |  |  |
| COPD | 91(23.1) | 12(4.3) | <0.001 |
| Coronary artery disease | 26(6.6) | 45(16.0) | <0.001 |
| Hypertension | 217(55.1) | 75(26.7) | <0.001 |
| Cholelithiasis | 4(1.0) | 1(0.4) | 0.828 |
| Malignant tumor | 26(6.6) | 17(6.0) | 0.898 |
| Diabetes mellitus | 76(19.3) | 108(38.4) | <0.001 |
| Vital signs |  |  |  |
| Respiratory rate (/min) | 21(17-27) | 21(18-27) | 0.483 |
| Heart rate (/min) | 101(85-118) | 105(87-120) | 0.199 |
| Systolic blood pressure (mmHg) | 123(107-143) | 116 (114-118) | <0.001 |
| Diastolic blood pressure (mmHg) | 70 (58-84) | 61 (59-62) | <0.001 |
| SOFA | 6(3-10) | 7(3-10) | 0.181 |
| Laboratory data |  |  |  |
| Red blood cell (*10^12^) | 3.79(3.09-4.43) | 3.91(3.31-4.48) | 0.043 |
| White blood cell (*10^9^) | 13.8(9.2-19.2) | 12.5(8.7-18.1) | 0.079 |
| Platelet (*10^9^) | 214(155-309) | 198 (148-275) | 0.063 |
| Neutrophil (*10^9^) | 10.7(7.1-14.8) | 10.6(7.2-15.6) | 0.811 |
| Lymphocyte (*10^9^) | 1.13(0.72-1.38) | 1.11(0.49-1.45) | 0.611 |
| Hemoglobin (g/L) | 113(93-135) | 117(102-138) | 0.001 |
| Hematocrit (%) | 35(29-40) | 36(31-41) | 0.031 |
| Total bilirubin (mg/dL) | 1.1(0.5-2.8) | 1.0(0.5-2.5) | 0.533 |
| Alanine aminotransferase (U/L) | 48(24-158) | 51(25-131) | 0.864 |
| Aspartate aminotransferase (U/L) | 75(34-202) | 75(34-181) | 0.955 |
| Lactate dehydrogenase (U/L) | 505(285-601) | 361(242-518) | <0.001 |
| Sodium (mmol/L) | 138(135-141) | 138(134-141) | 0.895 |
| Potassium (mmol/L) | 4.2(3.8-4.6) | 4.1(3.7-4.7) | 0.619 |
| Chloride(mmol/L) | 103(99-107) | 103(99-108) | 0.564 |
| Calcium (mmol/L) | 2.03(1.88-2.15) | 2.00(1.88-2.15) | 0.691 |
| Glucose (mmol/L) | 7.8(6.1-10.9) | 7.4(5.8-10.6) | 0.656 |
| Creatinine (mg/dL) | 1.1(0.8-2.0) | 1.1(0.8-1.9) | 0.608 |
| Blood urea nitrogen (mmol/L) | 21(13-34) | 21(12-33) | 0.567 |
| Albumin (g/dL) | 3.0(2.6-3.3) | 2.9(2.5-3.3) | 0.354 |
| Triglyceride (mg/dL) | 214(214-214) | 194(150-263) | <0.001 |
| Amylase (U/L) | 160(114-190) | 168 (112-222) | <0.001 |
| International normalized  ratio (seconds) | 1.3(1.2-1.5) | 1.3(1.1-2.0) | 0.968 |
| Prothrombin time (seconds) | 14.7(13.1-17.0) | 14.4(13.2-16.4) | 0.409 |
| Activated partial thromboplastin time (seconds) | 30.9 (26.9-35.9) | 30.9(27.0-35.1) | 0.559 |
| Invasive ventilator | 197(50.0) | 189(67.3) | <0.001 |
| Vasopressor | 146(37.1) | 118(42.0) | 0.224 |

**Abbreviations**: COPD, Chronic obstructive pulmonary disease; SOFA, Sequential Organ Failure Assessment.
